# Cerebral Edema in Chronic Mountain Sickness: a New Finding

**DOI:** 10.1038/srep43224

**Published:** 2017-02-24

**Authors:** Haihua Bao, Duoyao Wang, Xipeng Zhao, Youshen Wu, Guixiu Yin, Li Meng, Fangfang Wang, Lan Ma, Peter Hackett, Ri-Li Ge

**Affiliations:** 1 Department of Medical Imaging Center, Affiliated Hospital of Qinghai University, Xining, Qinghai, China; 2 Research Center for High Altitude Medicine, Qinghai University, Xining, Qinghai, China; 3 Institute for Altitude Medicine, Altitude Research Center, University of Colorado Denver School of Medicine, Telluride, CO, USA

## Abstract

We observed patients with chronic mountain sickness (CMS) in our clinic who developed progressive neurological deterioration (encephalopathy) and we wished to investigate this. We studied nine such CMS patients, and compared them to 21 CMS patients without encephalopathy, and to 15 healthy control subjects without CMS. All 45 subjects lived permanently at 3200–4000 m. Measurements at 2260 m included CMS symptom score, multi-slice CT, perfusion CT, pulse oximetry (SpO_2_%), and hemoglobin concentration (Hb). One patient had MRI imaging but not CT; 5 had CSF pressure measurements. CMS subjects had lower SpO_2,_ higher Hb, higher brain blood density, lower mean cerebral blood flow (CBF), and significant cerebral circulatory delay compared to controls. The nine CMS subjects with neurological deterioration showed diffuse cerebral edema on imaging and more deranged cerebral hemodynamics. CSF pressure was elevated in those with edema. We conclude that cerebral edema, a previously unrecognized complication, may develop in CMS patients and cause encephalopathy. Contributing factors appear to be exaggerated polycythemia and hypoxemia, and lower and sluggish CBF compared to CMS patients without cerebral edema; but what triggers this complication is unknown. Recognition and treatment of this serious complication will help reduce morbidity and mortality from CMS.

Chronic Mountain Sickness (CMS) is an illness of high-altitude residents, defined by exaggerated hypoxemia and excessive erythrocytosis in the absence of lung disease or other causes of polycythemia; it resolves with descent to a lower altitude, and recurs with relocation back to high altitude (>3000 m)^1^,^2^. Prevalence of CMS in high-altitude populations varies from 1.2% to 33%, depending on altitude of residence and ethnicity, with Han Chinese much more susceptible than Tibetans, who have genetically adapted to high altitude over many millennia^1^. Characteristic symptoms of CMS include fatigue, headache, dizziness, sleep disturbances, dyspnea, localized cyanosis, muscle and joint pain, and problems with concentration and memory^1^,^2^. Patients with CMS may also develop serious complications of pulmonary hypertension and right heart failure, the most common cause of death. The neurological symptoms are attributed to both hypoxemia and reduced cerebral blood flow from high blood viscosity. Supporting the latter idea, bloodletting of 650 to 1300 ml improves CNS symptoms^3^. To date, the only neurological complications reported have been cerebral thrombosis and infarct.

We observed in our clinic CMS patients who presented with unusual or more pronounced neurological symptoms than typical, including vomiting, lethargy, severe lassitude, weakness, and some with ataxia. These symptoms and findings are similar to acute high-altitude cerebral edema (HACE)^4^, and we wondered if these patients might have developed cerebral edema, which has not previously been reported in CMS. The purpose of this study then was to determine if indeed cerebral edema were present in these patients and if so, what factors might explain it.

## Results

### Clinical

The general characteristics of CMS and control subjects are shown in Table 1. The two groups were similar in terms of age and body weight, while the CMS group had higher Hb and Hct, and lower SpO_2_% than the control group. CMS score was of course higher in the CMS group. All nine patients with encephalopathy were admitted to hospital; this was unnecessary for the others, who were treated in the outpatient clinic. In the five who had lumbar puncture, mean CSF pressure was 21.1 ± 1.4 mmHg on the first hospital day. CSF cell count, differential and chemistry analyses were all normal. Various treatments for the hospitalized patients with cerebral edema included acute therapy of cerebral edema with supplemental oxygen, dexamethasone, furosemide, and mannitol, and additional routine therapy for CMS with oxygen, salvia miltiorrhiza and ginkgo biloba. Following standard practice in China, these patients did not receive bloodletting. All patients recovered completely within 2–3 weeks. Six of the nine patients with CMS and encephalopathy returned to their high altitude homes in Tian Jun and Maduo area, with a program of regular check-ups and Tibetan herb medication. The remaining three stayed in the Xining area. The CMS patients without encephalopathy received standard care with oxygen and Tibetan herbs in Xining and continued with the herbs on return to home altitude, and also had scheduled follow-up.

### Brain imaging

**Figure 1:**
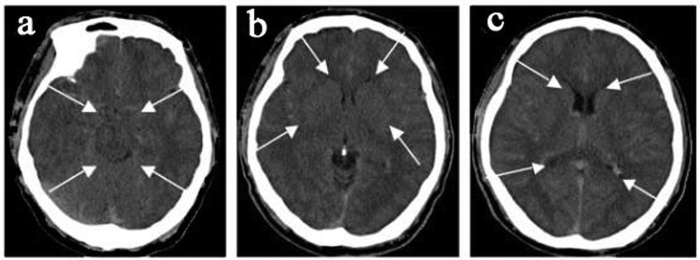
CMS patient, a 46-year-old male, hemoglobin 24.1 g/dl. CT shows diffuse cerebral edema with small ventricles (**c**), effaced sulci (**b**,**c**), and compromised cisterns (**a**,**b**).

**Figure 2:**
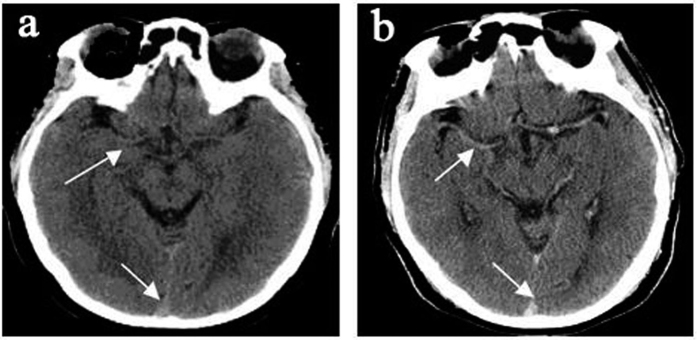
(**a**) Non-CMS subject, 38-year-old male, (**b**) CMS patient, 46-year-old male, showed higher density of bilateral middle cerebral arteries (arrow) and superior sagittal sinus (arrow), indicating higher blood density.

**Figure 3:**
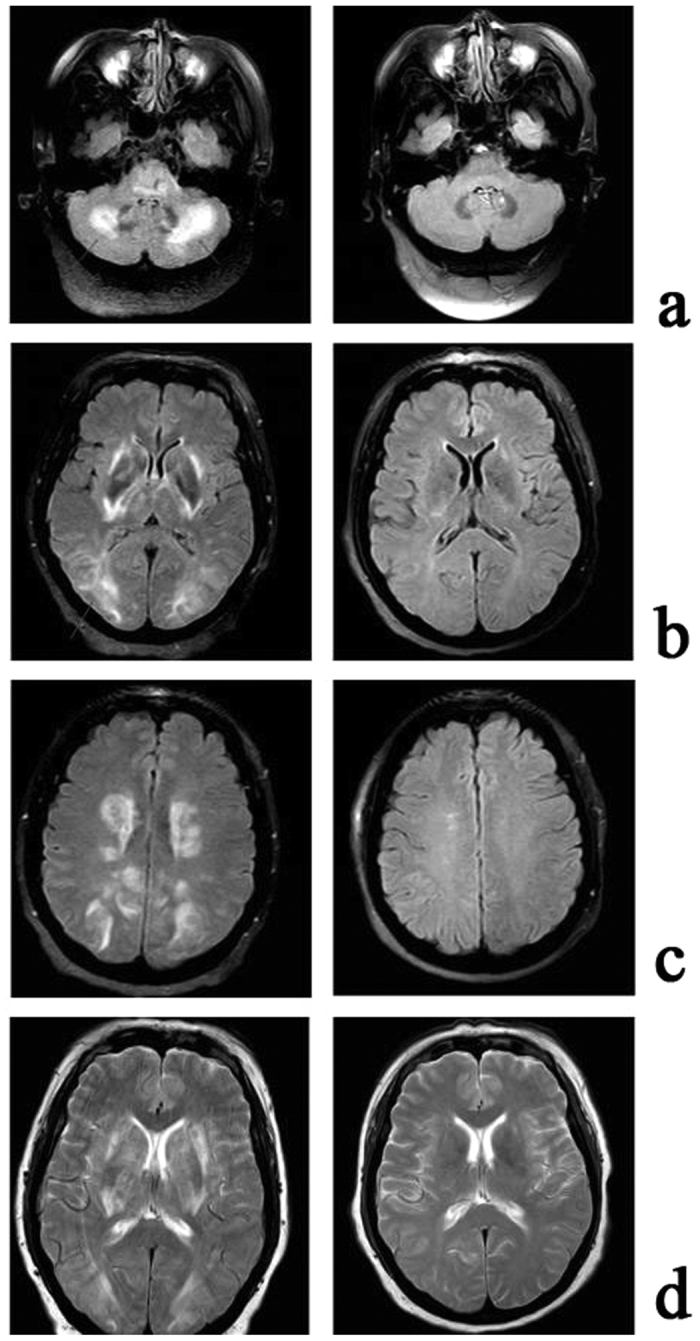
MRI of 51-year-old female CMS patient. Each panel shows acute (left) and recovery (right) images. Note markedly increased T_2_ signal in cerebellar hemispheres (**a**), internal capsule, parietal and occipital lobes (**b**–**d**). Abnormal signal disappeared two months later, with the patient fully recovered.

All 45 subjects had brain imaging; of the nine hospitalized patients with encephalopathy eight had CT and one had MRI. CT imaging in the hospitalized group showed diffuse cerebral edema with small ventricles and effaced sulci, as exemplified in Fig. 1. Two of these patients in addition to edema had multiple old cerebral infarcts, and one had chronic thrombosis of the superior sagittal sinus. The brain blood density (CT value of Hounsfield Units, HU) of the bilateral middle cerebral arteries and the superior sagittal sinus was greater in CMS compared to non-CMS groups (Table 1 and Fig. 2). The MRI was obtained in a patient who did not have CT. This 51-year-old female Han Chinese with CMS had resided at 3800 m for 15 years, suffering from headache, dizziness, fatigue, poor appetite, sleepiness, mood changes, and loss of memory for many years. Ten days prior to admission, she developed severe headache, vomiting, irritability, lethargy, and ataxia. Hemoglobin was 24.1 g/dl on admission and 15.4 g/dl after two-month’s treatment that included staying at the lower altitude, as well as oxygen, dexamethasone, and furosemide acutely, followed by Tibetan herbs. CSF pressure was 22.5 mmHg on admission. Initial MRI (Fig. 3) showed markedly increased T_2_ signal in cerebellar hemispheres, internal capsule, and parietal and occipital lobes that resolved after recovery two months later (Fig. 3). With recovery, apparent diffusion coefficient (ADC) values decreased in the cerebellar hemispheres from 1.26 ± 0.1 to 0.70 ± 0.06 (10^−3^ mm^2^/s) (Fig. 3a left& right), and from 0.97 ± 0.07 to 0.75 ± 0.02(10^−3^mm^2^/s) (Fig. 3b left & right) in other regions.

### CTP images

**Figure 4:**
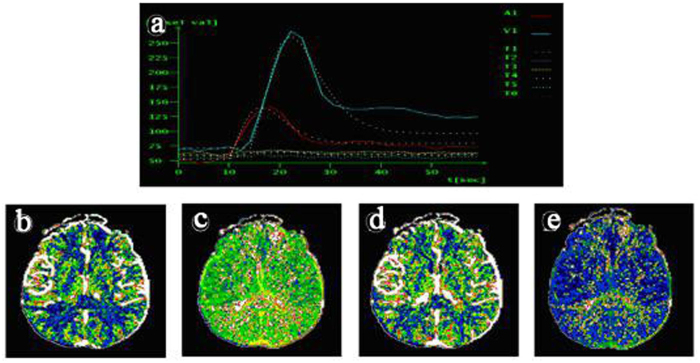
Non-CMS subject, 43-year-old male. Brain CT perfusion images, including (**a**) time delay curve (TDC) map, (**b**) cerebral blood flow (CBF), (**c**) time-to-peak (TTP), (**d**) cerebral blood volume (CBV), and (**e**) mean transit time (MTT). On TDC curve, mean peak time for superior sagittal sinus and bilateral MCA arterial perfusion was 22 seconds (blue line) and 18 seconds (red line), respectively.

**Figure 5:**
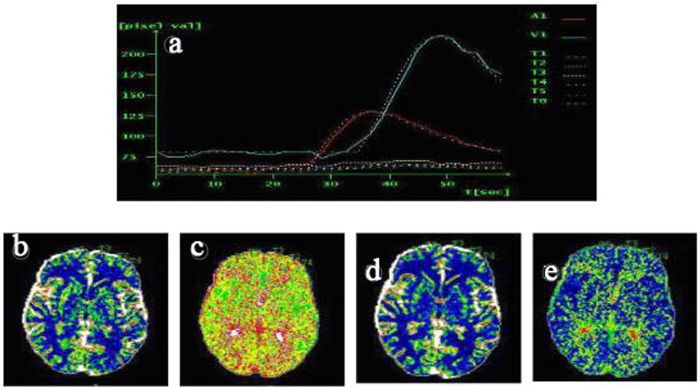
CMS patient, 28-year-old male. Brain CT perfusion images, including (**a**) time delay curve (TDC) map, (**b**) cerebral blood flow (CBF), (**c**) time-to-peak (TTP), (**d**) cerebral blood volume (CBV), and (**e**) mean transit time (MTT). On the TDC curve, mean peak time of superior sagittal sinus and bilateral middle cerebral artery perfusion was 50 seconds (blue line) and 35 seconds (red line), respectively, which was very prolonged compared to the non-CMS group (see Table 1).

CT perfusion parameters included mean transit time (MTT), time-to-peak perfusion (TTP), cerebral blood flow (CBF), cerebral blood volume (CBV), and time delay curve (TDC) map. MTT is time between arterial inflow and venous outflow, thus reflecting the state of blood flow in the entire capillary network. TTP is the time contrast takes to achieve maximum enhancement (HU value) in the selected region of interest (ROI). Table 1 shows comparison of these parameters in grey and white matter between groups. Grey matter CBF in CMS subjects was significantly lower than in controls (p < 0.001), and both TTP and MTT were significantly prolonged in the CMS group grey matter. In white matter, MTT was delayed in CMS compared to non-CMS groups. TDC data are shown in Table 1, Figs 4 and 5; the mean peak time in superior sagittal sinus and bilateral middle cerebral arteries in CMS patients (Fig. 5a) was significantly longer than in control subjects (Fig. 4a).

A comparison of CMS patients with cerebral edema to those CMS patients without edema is provided in Table 2. Cerebral edema patients had higher CMS score and hemoglobin, and lower SpO_2_%. Perfusion scanning revealed lower CBF and greater circulatory delay in those CMS patients with cerebral edema compared to CMS patients without cerebral edema.

## Discussion

The surprising finding of this study is that cerebral edema appears to be a complication of Chronic Mountain Sickness. To our knowledge, this is the first report describing cerebral edema in CMS, and given the high incidence of this problem in mountainous regions of the world, this finding is clinically valuable. The second new finding is significantly reduced cerebral blood flow, and cerebral circulatory delay in patients with Chronic Mountain Sickness. Exactly how these two findings are related is unclear.

This was not a study of incidence, and we do not know how many patients with CMS actually develop cerebral edema. Regardless, it is clear that cerebral edema occurs in CMS. These patients would most likely have died without treatment. Indeed, patients with CMS may have died from cerebral edema, but no reports of necropsy in CMS exist from this region to verify this, and the only few autopsy reports available from South America showed death from heart failure, with mention of “congestion of the brain”^5^. Our report should alert physicians to the possibility of potentially fatal cerebral edema in CMS patients. Early recognition and treatment might prevent complications such as cerebral infarctions and thrombosis, as well as progression to death.

Treatment of CMS with descent to lower altitude, supplemental oxygen and Tibetan herbs is the standard in this part of China, and was successful. However, controlled trials of the herbs are lacking, and whether descent and oxygen are sufficient is unknown. In other countries, notably in Peru, bloodletting is sometimes used, and controlled trials have demonstrated subjective benefit but little physiological or measurable clinical benefit^1^. The practice has not been adopted in China for concerns of acute anemia, and rebound polycythemia afterwards. Recent work has shown that acetazolamide and ACE inhibitors are useful in treating CMS in patients who remain at their home altitude. To our knowledge, these medications have not been introduced to China, and a trial comparing them to the Tibetan herbs would be informative. In this study, we were concerned with immediate treatment of life-threatening cerebral edema, and used diuretics in addition to other therapies. We must emphasize that diuretics are best avoided in CMS, since dehydration will aggravate polycythemia and microcirculatory sludging. There were no apparent complications of diuretic use in these patients with CMS and cerebral edema, and we used them in low doses and for short duration. Whether bloodletting is a valuable adjunct in those with CMS and cerebral edema, and might improve safety, awaits further study.

The mechanism of brain edema in this setting is unclear. While all CMS patients had greater hypoxemia, polycythemia, reduced CBF and cerebral circulatory delay compared to healthy high-altitude residents, these factors were all exaggerated in those with brain edema, raising the possibility that at least in some persons, severe CMS per se can lead to cerebral edema. The clinical presentation and response to treatment were comparable to acute high altitude cerebral edema (HACE), and perhaps mechanisms are similar. One possibility is that a relatively acute drop in cerebral tissue oxygenation in these patients mimics acute ascent to altitude. We theorize that in CMS the combination of low cerebral blood flow and low arterial oxygen pressure results in a precarious low flow/low O2 state resulting in marginal cerebral tissue oxygenation despite the high arterial oxygen content due to the very high hemoglobin. Under these circumstances, acute reductions in arterial oxygen pressure, sleep disordered breathing, carbon monoxide exposure, blood pressure changes, dehydration, and ascent to higher altitude are all factors that could act as a “tipping point” for acute inadequate tissue oxygenation similar to acute ascent to altitude, and subsequent “high altitude” cerebral edema.

Specific mechanisms suggested for acute HACE include alteration of cerebral autoregulation, endothelial dysfunction, leaking of the blood brain barrier with extracellular vasogenic edema, and eventually cytotoxic edema^4^,^6^,^7^,^8^. In addition to these mechanical factors, cytokines, altered signaling, HIF-induced gene regulation, and other mediators of permeability may also play a role^9^. These same general mechanisms may participate in the cerebral edema in our CMS population, but have not been investigated.

To our knowledge, measurements of CBF in CMS are limited, and there are no previous CT perfusion measurements in CMS patients, or investigations of cerebral hemodynamics, except by transcranial Doppler (TCD). The finding of reduced CBF is consistent with baseline measurements in earlier studies with TCD, and consistent with the established association of polycythemia and reduced CBF. We also found a continuum of reduction in CBF with increasing severity of CMS across all subjects, not just those with edema, which might argue against the possibility that the reduced CBF in the most ill was due to the edema itself. However, CBF in brain edema is expected to be low. Previous studies have demonstrated loss of cerebral autoregulation in healthy Sherpa highlanders in Nepal^10^, but this has not been assessed in CMS. Sun *et al*. found a lack of compensatory increase in CBF during sleep hypoxemia in CMS patients in Lhasa (7 Han, 1 Tibetan), measured by carotid blood flow velocity^11^. Appenzeller and colleagues studied eight Peruvian men, four with CMS, and found no cerebral vasodilatation in response to NO, measured by TCD^12^, but no differences between CMS and non-CMS highlanders. They suggested that the cerebral vascular response to NO indicates fitness for life at high altitude, and that this is lacking in Andean natives, although not specifically in CMS. Further studies of cerebral vascular reactivity will be necessary to confirm that CMS is associated with blunted cerebral vascular responses to hypoxemia or external stimuli, and to what extent CMS patents with cerebral edema differ from CMS patients without edema. While animal models have been useful for understanding acute hypoxic cerebral edema, we are not aware of an animal model that develops cerebral edema with chronic hypoxia.

This work has limitations. We lack complete clinical information, such as exact duration of symptoms, follow-up months later, and other possible comorbidities. However, we did follow these patients for the duration of their treatment and stay in Xing, and most other illnesses were ruled out by careful physical examination and appropriate laboratory and imaging studies, as well as the course of their recovery. We did not evaluate our subjects carefully for smoking history. While normal spirometry rules out significant COPD, there may be some effect of smoking even with normal spirometry. We did not obtain history of carbon monoxide exposure, but removal of the patients from their home environment to Xining would have resolved CO exposure, and these patients were the only ill persons from their families, making a home dwelling CO exposure unlikely. Only one MRI was obtained, and more MRI data would help evaluate the nature of the brain edema. MRI might also enable early diagnosis and therefore early treatment. Future research should include development of an animal model of cerebral edema in chronic hypoxia to help elucidate the mechanisms. Prospective clinical studies could determine whether some patients with CMS have subclinical edema and would help elucidate risk factors and possible triggers for developing cerebral edema. Bloodletting, acetazolamide and ACE inhibitors should also be investigated.

In conclusion, some patients with chronic mountain sickness are at risk of developing cerebral edema. This is a newly recognized complication of CMS (Cerebral Edema of Chronic Mountain Sickness). Clinical and CT perfusion data support the idea that exaggerated severe chronic hypoxemia and excessive polycythemia in patients with CMS are contributing factors, but exact mechanisms are unknown. Recognition and treatment of this serious complication will help reduce morbidity and mortality from CMS.

## Methods

### Ethical approval

This study conformed to the standards of the Declaration of Helsinki for medical research involving human subjects. All subjects signed the informed consent and all experimental protocols were approved by the ethics committee of the Affiliated Hospital of Qinghai University.

### Subjects and symptom score

Our 45 subjects included 30 CMS patients (one female) and 15 non-CMS male subjects, all permanent residents of the Qinghai–Tibetan plateau (3200 m to 4000 m). CMS subjects were recruited over the course of one year from the high-altitude medical clinic of the Affiliated Hospital of Qinghai University in Xining (2260 m), where they had been referred for medical evaluation. One experienced physician (GRL) evaluated all subjects in this convenience sample. 28 of 30 CMS subjects were Han Chinese, as were all control subjects without CMS; two CMS patients were Tibetan. Nine CMS patients had developed encephalopathy over one to two weeks prior to their evaluation. These nine patients were admitted to hospital and studied within 24 hours, while CMS patients without CNS deterioration were studied as outpatients within two days after their arrival. The non-CMS control subjects, who had also presented for medical evaluation for various non-neurological complaints, were studied two days after they descended to Xining from their home altitude. We assessed presence and severity of CMS by international criteria (Qinghai CMS Score), which we described previously^2^,^10^. The score obtained in Xining reflected the patients’ condition at their home altitude, since symptoms did not change acutely with descent. The score includes symptoms of headache, breathlessness, palpitations, sleep disturbance, cyanosis, tinnitus, paresthesia, and dilatation of veins. For this study, we used the presence of facial telangiectasia as an indication of venous dilatation. In addition, for diagnosis of CMS, hemoglobin concentration must be ≥21 g/dl in males and ≥19 g/dl in females. The nine hospitalized patients in addition to the general symptoms of CMS had nausea or vomiting, mood changes, lethargy, drowsiness, and ataxia. None of these patients had notable brain dysfunction previously. These symptoms had gradually increased over 1–2 weeks despite treatment with salvia miltiorrhiza and rhodiola sachalinesis at the local clinics, prompting their referral to Xining. The other CMS patients referred to the clinic had persistent symptoms of CMS, but without CNS progression. Subjects were excluded if they had: (1) chronic obstructive pulmonary disease, (2) asthma, (3) congenital heart disease, (4) a documented neurological disorder, or (5) a past history of head injury with loss of consciousness.

### Clinical Tests

We measured height, weight, blood pressure, and heart rate by standard methods. Hb concentration and Hct were determined from venous blood samples using the Mindray Hematology Analyzer (BC-2300, Shenzhen, China). We used a pulse oximeter (Ohmeda 3700 Pulse Oximeter, Datex-Ohmeda, Boulder, Colorado, USA) to record SpO_2_%. Pulmonary function tests were performed using a standard electric spirometer controlled by a microcomputer (Transfer Screen II: Jaeger; Wuirzburg, Germany). Lung volumes and maximal flow-volume loop were recorded and analyzed by the Jaeger system. CSF pressure was obtained by lumbar puncture and standard manometer, measured lying flat in the decubitus position. CSF analysis included standard cell counts and chemistries.

### Plain CT scan and perfusion CT imaging

We used a 16-detector CT scanner (Philips Brilliance, Philips Medical System, Netherlands) for plain and perfusion CT scans. Slice thickness was 6 mm, acquisition matrix 128*128*. Additionally, blood density was quantified by measuring CT attenuation values (HU) in regions of interest (ROIs) that contained the bilateral middle cerebral artery and superior sagittal sinus, using standard circular ROI measuring 2 mm^2^. All ROI measurements were made by an independent observer blinded to both clinical and laboratory data.

#### CT perfusion (CTP)

Unenhanced CT scan was immediately followed by CTP scan, using a dynamic first-pass bolus-tracking methodology. Scan parameters were 6 mm slice thickness, 120 kVp, 150 mAs, matrix 512*512, total scan time 45 s during the automatic injection of 50 ml of non-ionic contrast agent (Ultravist 300, Schering, Berlin, Germany) containing 300 mg of iodine/ml, at a flow rate of 5 ml/s into the right median cubital vein. 120 images were analyzed for CTP parameters in each subject. CTP maps were created with CTP prototype software (Philips Medical Systems, Best, Netherlands). We selected the anterior cerebral artery as arterial input function (AIF) and the superior sagittal sinus as venous output function (VOF). Six ROI were selected in white matter and grey matter (frontal lobe, temporal lobe and occipital lobe). Two experienced neuroradiologists blinded to all other data analyzed images independently. One patient had brain magnetic resonance images rather than CT scan (MRI; Philips Achieva TX 3T), on admission and after treatment two months later. Apparent diffusion coefficient (ADC) values were calculated precisely on the T2 maps. MR sequences included T1WI, T2WI, DWI, and FLAIR.

### Statistical analysis

Data are expressed as mean and standard deviation (SD). Comparisons between CMS and non-CMS groups and between cerebral edema and non-cerebral edema CMS subjects were made by the Fisher protected least significant difference test, and a student unpaired t-test. A p value < 0·05 was chosen to represent significance. We used SPSS software (Version 13·0; Chicago IL, USA).

## Additional Information

**How to cite this article**: Bao, H. *et al*. Cerebral Edema in Chronic Mountain Sickness: a New Finding. *Sci. Rep.* **7**, 43224; doi: 10.1038/srep43224 (2017).

**Publisher's note:** Springer Nature remains neutral with regard to jurisdictional claims in published maps and institutional affiliations.

## Figures and Tables

**Table 1:** Clinical variables and CT perfusion parameters in CMS and non-CMS groups.

Variables	CMS (N = 30)	Non-CMS (N = 15)	P value
Age, years	43.5 ± 7.30	42.3 ± 3.5	0.330
Body-mass index (kg/cm^2^)	24.3 ± 1.3	24.6 ± 1.6	0.332
Hemoglobin (g/dl)	23.1 ± 1.6	14.6 ± 6.9	<0.001
Hematocrit (%)	70.9 ± 4.2	45.5 ± 5.4	<0.001
HR (beats/min)	83.7 ± 14.1	77.6 ± 13.9	0.221
SpO_2_ (%)	85.1 ± 4.8	93.1 ± 2.8%	<0.001
Forced Vital Capacity, FVC (l)	4.23 ± 0.67	4.41 ± 0.77	0.136
FEV1/FVC %	87.69 ± 5.43	88.31 ± 5.24	0.491
FEF 25–75% (l/s)	3.30 ± 0.64	3.18 ± 0.59	0.261
CMS score	13 [8–20]	1 [1–2]	<0.0001
CT value of SSS (HU)	55.91 ± 4.61	46.39 ± 2.76	<0.01
CT value of BMCA (HU)	51.29 ± 5.13	38.83 ± 3.29	<0.01
ACA-TDC (s)	25.50 ± 5.20	19.87 ± 2.26	<0.05
SSS-TDC (s)	35.92 ± 5.68	26.80 ± 3.69	<0.001
**White Matter**			
CBF [ml/(100 ml/min)]	15.6 ± 3.4	17.3 ± 3.6	0.270
TTP (s)	17.4 ± 2.6	15.1 ± 3.3	0.121
CBV (ml/100 g)	2.91 ± 1.1	3.07 ± 0.9	0.453
MTT (S)	10.7 ± 2.0	6.69 ± 2.3	0.004
**Grey Matter**			
CBF [ml/(100 ml/min)]	30.4 ± 4.8	40.5 ± 3.9	0.001
TTP (s)	14.0 ± 2.5	11.0 ± 2.3	0.017
CBV (ml/100 g)	4.60 ± 1.0	4.47 ± 0.9	0.358
MTT (s)	6.51 ± 1.6	3.97 ± 0.7	0.004

Data expressed as mean ± standard deviation, FEV_1_%, percent of forced expiratory volume in 1 second; FEF_25–75%_, mean forced expiratory flow during half of the forced vital capacity; SSS, superior sagittal sinus; BMCA, bilateral middle cerebral artery; ACA-TDC, anterior cerebral artery time delay curve; SSS-TDC, superior sagittal sinus time delay curve; CBF, cerebral blood flow; TTP, time-to-peak; CBV, cerebral blood volume; MTT, mean transit time.

**Table 2:** Comparison of clinical variables and CT perfusion parameters in CMS-CE and CMS-nonCE.

Variable	CMS-CE (n = 9)*	CMS-nonCE (n = 21)	*P* value
Age (yr)	42.6 ± 8.1	43.6 ± 6.8	0.3916
BMI (kg/cm^2^)	24.3 ± 1.3	24.1 ± 1.2	0.766
Hb (g/dl)	25.5 ± 0.7	23.1 ± 1.4	<0.0001
Hct (%)	74.1 ± 3.5	66.5 ± 5.1	0.0105
HR (beats/min)	80.7 ± 4.2	77.2 ± 8.3	0.1346
SpO_2_ (%)	80.1 ± 4.2	88.6 ± 4.5	0.003
CMS score	15.6 ± 3.6	10.3 ± 32	0.017
CSF pressure (mm Hg)	21.1 ± 1.4	—	
CT-SSS (HU)	58.3 ± 2.7	56.4 ± 5.3	0.184
CT-BMCA(HU)	50.7 ± 3.3	48.4 ± 3.4	0.382
**White Matter**			
CBF (ml/100 g/min)	15.4 ± 3.2	17.4 ± 4.8	0.04
TTP (s)	18.4 ± 4.8	18.1 ± 3.2	0.700
CBV (ml/100 g)	3.06 ± 0.9	2.89 ± 1.2	0.701
MTT (S)	8.91 ± 1.4	9.3 ± 2.4	0.182
**Grey Matter**			
CBF (ml/100 g/min)	28.9 ± 4.1	35.5 ± 3.2	0.017
TTP (s)	16.9 ± 7.5	16.3 ± 2.1	0.585
CBV (ml/100 g)	4.84 ± 1.0	4.17 ± 0.7	0.103
MTT (s)	5.58 ± 0.8	5.12 ± 0.9	0.386

Data expressed as mean ± SD. CE, cerebral edema; SSS, superior sagittal sinus; BMCA, bilateral middle cerebral artery.; CBF, cerebral blood flow; TTP, time-to-peak; CBV, cerebral blood volume; MTT, mean transit time. Attention: only 5 of the CMS with edema group have CSF value. ^*^Nine patients with CE, but n = 8 for 8 CT values.
